# A Standardized Protocol for Analyzing Masticatory Muscle Activity at Different Levels of Mouth Opening Using Electromagnetic Articulography and Surface Electromyography: A Proof-of-Concept Study

**DOI:** 10.3390/bioengineering12080811

**Published:** 2025-07-28

**Authors:** Franco Marinelli, Camila Venegas-Ocampo, Josefa Alarcón-Apablaza, Joaquín Ruiz, Gastón Schlotthauer, Rosemarie Schneider, Ramón Fuentes

**Affiliations:** 1 Research Centre in Dental Sciences of the Universidad de la Frontera (CICO-UFRO), Facultad de Odontología, Universidad de La Frontera, Temuco 4811230, Chile; franco.marinelli@ufrontera.cl (F.M.); camilabelen.venegas@ufrontera.cl (C.V.-O.); josefa.alarcon@ufrontera.cl (J.A.-A.); r.schneider01@ufromail.cl (R.S.); 2 Facultad de Ciencias de la Salud, Universidad Autónoma de Chile, Temuco 4810101, Chile; 3 Research Center in Health Sciences, Universidad Adventista de Chile, Chillán 3780000, Chile; 4 Doctoral Program in Morphological Sciences, Faculty of Medicine, Universidad de La Frontera, Temuco 4810101, Chile; 5 Laboratory of Signals and Nonlinear Dynamics, Research and Development Institute in Bioengineering and Bioinformatics (IBB), Consejo Nacional de Investigaciones Científicas y Técnicas—Universidad Nacional de Entre Ríos (CONICET—UNER), Oro Verde 3100, Argentina; joaquin.ruiz@uner.edu.ar (J.R.); gaston.schlotthauer@uner.edu.ar (G.S.)

**Keywords:** oral physiology, human movement analysis, standardization

## Abstract

The study of muscle activity as a function of vertical dimension has been extensively developed in the field of oral physiology. It involves asking subjects to open their mouths to a predetermined distance and then recording muscle activity in that position. Most studies perform this without accounting for physiological differences among patients. The objective of this study is to present a protocol for recording muscle activity at various mouth-opening levels using electromagnetic articulography (EMA) and surface electromyography (sEMG), normalizing opening degrees and muscle activity. Muscle activity recordings were obtained in the position of maximum intercuspation and maximum mouth opening. Based on these recordings, the position corresponding to 5–50% of maximum opening was calculated. EMA and sEMG recordings were performed at these levels. Muscle activity during maximum voluntary clenching was recorded and used to normalize the previous data. In all cases, three 5-second recordings were obtained. The analysis of muscle activity using EMA and sEMG did not present any complications. A slight difference was observed between the intended percentage of mouth opening and the actual percentage achieved. The method described in this study is a tool that allows for the analysis of muscle activity at various mouth-opening levels in a way that has not been previously explored in the literature.

## 1. Introduction

Electromyography is a widely used technique in dentistry as a research method [1]. It is applied both in physiology and pathology, as it aims to identify patterns that describe healthy individuals and to determine whether these patterns change in patients with certain pathologic conditions [2]. There is ongoing debate regarding how muscle activity behaves at different levels of oral opening. In early studies, Garnick and Ramfjord identified a resting range between 3 and 14 mm of opening for the masseter and temporal muscles, and thus proposed a resting range rather than a single point [3]. Gross et al. reached similar conclusions [4]. On the other hand, it has been observed that muscle activity progressively decreases as oral opening increases, reaching a minimum, and then rises again toward maximum mouth opening (MMO) [5]. While a point of minimum activity is described, it is generally found within a range of 8 to 11 mm of opening [6,7,8,9]. The variation in muscle activity seen with oral opening has had clinical implications for the design of occlusal splints. Manns et al. found that designing splints that guide the mandible toward a position of minimum electromyographic activity led to faster symptom improvement compared to thinner splints [10]. Currently, the most commonly used splints have a thickness of 3–5 mm [11]. However, there is still limited evidence that this type of treatment produces a significant clinical effect [12].

The study of muscular activity and mouth opening involves two techniques: one that records muscular activity and the other that records mouth opening. Muscular activity is generally recorded using the surface electromyography (sEMG) of the masseter and anterior temporal muscles, although there are studies that have used needle electromyography [5,13,14,15]. On the other hand, mouth opening has been evaluated using different methods. Since the system developed by Garnick and Ramfjord, several techniques have been developed [3,16]. These have ranged from mechanical systems using calipers to digital equipment based on ultrasound, cameras, kinesiography, and electromagnetic articulography (EMA) [17,18,19,20,21]. Once the techniques are defined, the subject is asked to open their mouth, starting from maximum intercuspation (MIC) to a determined position, potentially reaching MMO, and muscular activity is recorded.

These recording techniques can explain the variability in results, as maximum mouth opening ranges from 30 to 50 mm, so a specific opening represents a different position for each person [22,23]. Due to interindividual variability in the range of mouth opening, analyzing muscle activity at a fixed distance, for example, 40 mm, may correspond to the maximum opening for one subject, while representing only 70% of the full range for another. On the other hand, studies often overlook the normalization of the electromyographic signal, which is necessary for making comparisons between patients [24,25]. This has made it difficult to compare studies, as they often differ in the equipment used and the method of quantifying muscular activity, such as the average signal, the root mean square (RMS) value, the integrated EMG, among others [17,26,27,28]. Finally, the values obtained in each study can vary by orders of magnitude, ranging from 0.5–5 µV to 10–50 µV, further complicating the comparison between studies [5,7,8,29,30].

The aim of this work is to develop a protocol for recording muscular activity and mouth opening using sEMG and EMA, normalizing the degree of opening and muscular activity, which will allow for a comparable presentation of results between subjects and studies.

## 2. Materials and Methods

Four subjects participated in this study, including two 19- and 22-year-old males and two 19- and 20-year-old women, all with Class I occlusion and no signs or symptoms of TMD. A symptom questionnaire for TMD was applied to each subject at the Oral Physiology Laboratory of Universidad de La Frontera by a trained dentist (R.F.). This questionnaire was the Spanish version of the tool developed by the International Network for Orofacial Pain and Related Disorders Methodology [31]. The present study was conducted with the approval of the Scientific Ethics Committee of the Universidad de La Frontera (Record No. 123_24). The subjects were identified using the following NNLNN code, where the first two numbers indicate the subject’s identification number within the study, L is a letter representing the subject’s gender, and the last two numbers indicate the subject’s age.

For the mandibular position, EMA was used (AG501; Carstens Medizinelektronik GmbH, Bovenden, Germany). This technique is based on magnetic induction by a set of transmitting coils that induces a current in small receiving coils that act as motion sensors and are attached to the structure with the movement that is to be recorded. This current is used by the system to determine the position of the sensors in space [32]. This equipment allows for the real-time visualization of the sensor coordinates and provides the position of sensors in millimeters with an accuracy of 0.3 mm [33].

The motion sensor was placed on the interincisal line of the mandibular frontal incisors. To eliminate head movement, EMA features the Head Correction procedure. This requires reference sensors to be placed on the glabella and mastoid muscles. All sensors were attached to subjects using tissular adhesive (Epiglu^®^; Meyer-Haake GmbH, Ober-Mörlen, Germany).

sEMG was used to record muscle activity (EMG VIII; ArtOficio, Santiago de Chile, Chile), with a sampling frequency of 1311 Hz. Bipolar Ag/AgCl electrodes (H124SG, Kendall, Waukegan, IL, USA) were placed on the masseter and anterior temporal muscles over the right and left sides (RT, LT, RM, LM), with a center-to-center distance of 24 mm. To place the electrodes, subjects were asked to perform a maximal bite while the muscles were palpated to find the most prominent area. Once located, the electrodes were placed in this area following the direction of the muscle fibers. A comb-type band stop filter was applied to the sEMG signals to eliminate line noise (50 Hz) and its harmonics. The filter was implemented as a series of 4th-order high-pass filters with a bandwidth of 3 Hz and an attenuation of −4 dB in the bandwidth. The central frequencies of each filter were defined as fk = k50, for k = 1, 2, …, K, where K is the number of components or “teeth” of the comb filter, which are defined in the range (0, fs/2). It is important to note that the sEMG signal was resampled at 1350 Hz to ensure that the sampling frequency was an integer multiple of the fundamental frequency of the line noise. Therefore, the number of filters used to construct the comb filter was K = [⌊ (1350/2)/50⌋ = 13, where ⌊ ⌋ indicates the nearest lower integer. After filtering, the signal was resampled to the original frequency of 1311 Hz.

### 2.1. Normalization of Mouth Opening

To normalize mouth opening, the Z axis coordinates at maximum intercuspidation (*z_MIC_*) and maximum mouth opening (*z_MMO_*) were recorded using the EMA system. To obtain *z_MIC_*, the subject was asked to keep their teeth lightly in contact, while for *z_MMO_*, the subject was asked to open their mouth until the maximum extent was reached. Once this was achieved, the EMA and EMG recordings began. Five-second recordings were used, as seen in other similar studies, in order to reduce the session time and prevent subject fatigue [5,7,34]. Three 5-second recordings were made for both cases, and the average Z coordinate was obtained for each case. In each recording, the z-coordinate of the motion sensor observed in the EMA interface was recorded, and the average of the three recordings was calculated for both MIC and MMO. These average values were used as *z_MIC_* and *z_MMO_*_._

With these coordinates, the position (*z_i_*) that the subject should reach to obtain the desired percentage of opening (*∆_%_*) was determined using the following formula:

(1) $$z_{i}=\;z_{MIC}+\;{∆}_{\%}\;*\;(z_{MMO}-z_{MIC})$$

Once the corresponding coordinate for each percentage of opening was obtained, recordings were made for openings between 5 and 50% in 5% intervals.

For these recordings, the subject was asked to open their mouth while the Z coordinate of the motion sensor was visualized, until the desired position calculated using Equation (1) was reached. Once this was achieved, the subject was asked to maintain that position while the EMA and EMG recordings were made. Three 5-second recordings were taken for each case. For each recording, the average Z coordinate was obtained to determine the position reached.

In order to obtain the actual position reached during each recording, the average position over the entire 5-second recording was calculated. Then, the average of the three recordings was taken as the final value. These values were used to compare the intended percentage of mouth opening with the actual value reached. Based on this procedure, the *z_MIC_* and *z_MMO_* values were recalculated. The target coordinates corresponding to the desired percentage of mouth opening were then recalculated and compared with the values actually reached during the recordings. To assess the relationship between the intended position for each percentage of mouth opening and the actual position reached, the root mean square error (RMSE) was calculated.

### 2.2. Normalization of Muscular Activity

To normalize the muscular activity recordings, three recordings were made at maximum voluntary contraction (MVC). The subject was asked to clench their jaw for 5 s while the muscular activity was recorded. After each recording, a 2 min rest period was observed. For each recording, the root mean square value (*V_RMSi_*) was obtained. From the MVC recordings, the maximum value (*V_MVC_*) was obtained and used to calculate the normalized value for each recording (*V_%i_*) using the following equation:

(2) $$V_{\%i}=\;\frac{V_{RMSi}}{V_{MVC}}\;*\;100\%$$

Finally, the average was calculated from each set of the three normalized recordings. The data were processed with Malab (2020a, Version: 9.8.0.1323502; The MathWorks Inc., Natick, MA, USA).

## 3. Results

The values required for normalization were successfully obtained, as shown in Table 1.

**Figure 1:**
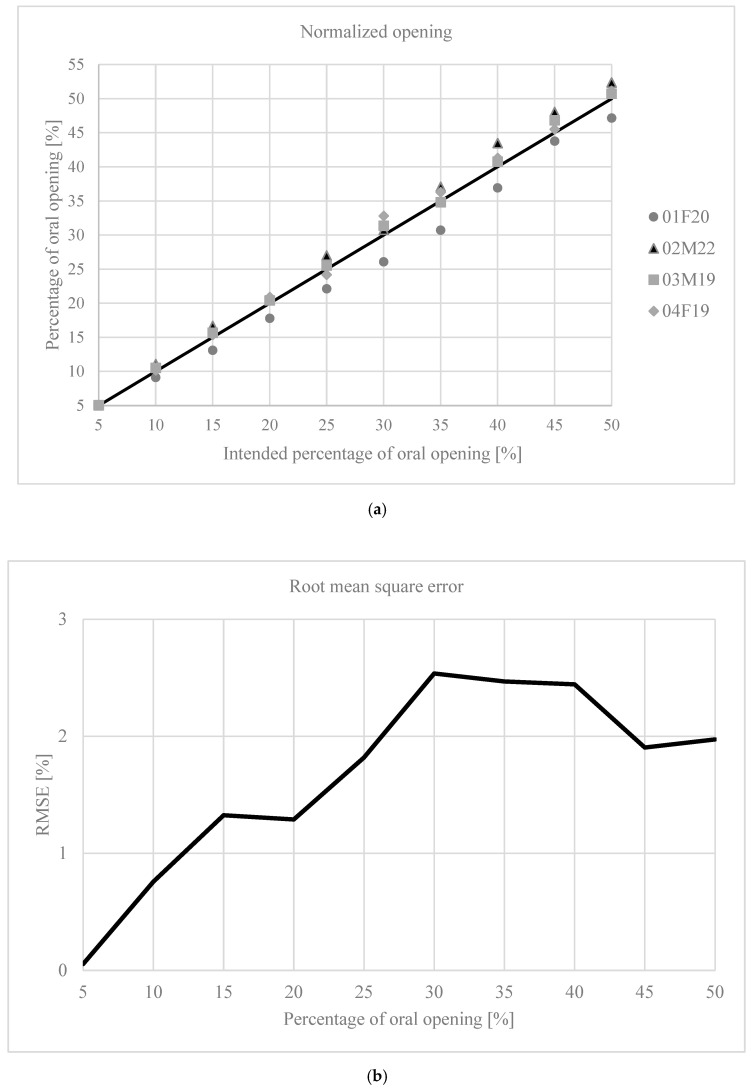
Mouth opening. (**a**) Intended normalized mouth opening and the actual opening achieved during the recording. The black line represents the ideal situation; (**b**) the RMSE for each degree of mouth opening.

**Figure 2:**
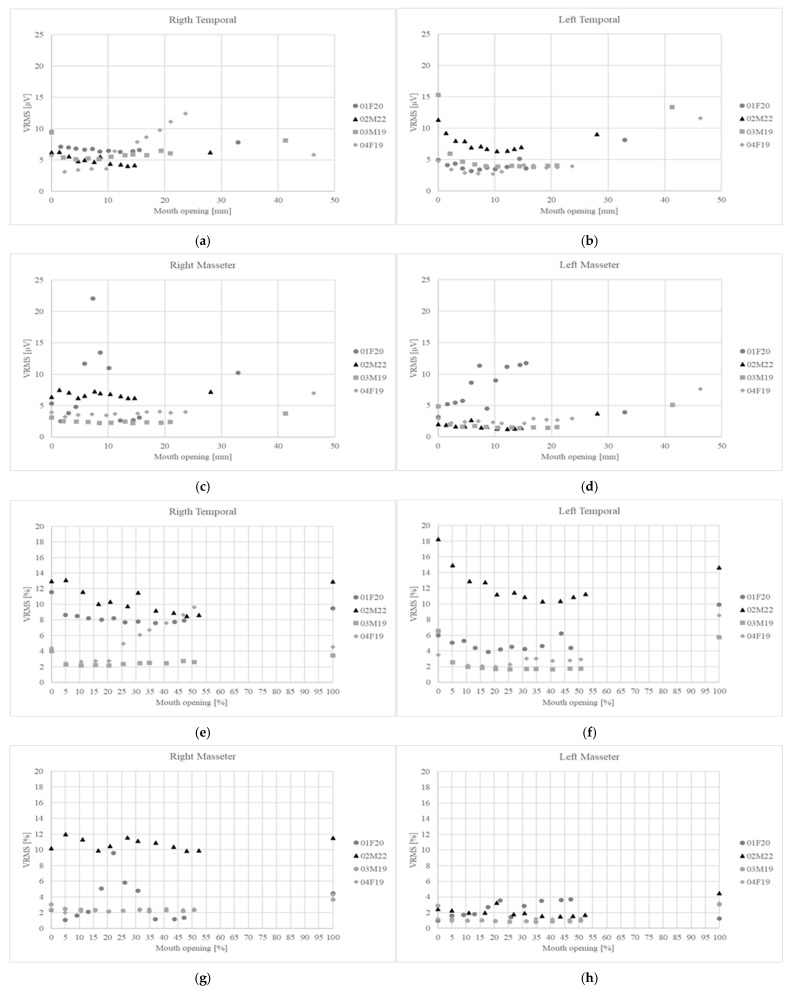
The variation in muscle activity as a function of mouth opening for the masseter and temporal muscles. (**a**–**d**) Absolute values; (**e**–**h**) normalized values.

Figure 1 shows the results corresponding to the normalization of oral opening. Figure 1a shows the percentage of opening reached by the subject at the time of measurement in relation to the intended target; the black line represents the ideal behavior. The root mean square error (RMSE) for this dataset was 1.83%. Figure 1b shows the RMSE for each opening level. Finally, Figure 2 shows the variation in muscle activity for the four muscles.

Figure 2a–d display the variation in muscle activity in absolute values, while Figure 2e–h show the same in normalized values.

## 4. Discussion

The MMOs of both subjects were 28 and 46 mm. This is consistent with what has been reported in the literature [22]. The values obtained for *V_MVC_* have also been found in other studies (Table 1) [26,28].

When observing the deviation in the relative mouth opening levels achieved compared to the ideal, it increases up to around 30% of opening (Figure 1a), where there is a maximum error of 2.5% (Figure 1b). In a range of 30–50 mm, this corresponds to a deviation of 0.75–1.25 mm. Studies that have analyzed mouth opening often use increments of this magnitude as well as larger increments [5,7,8,9,29,35,36,37]. Some studies have used step-wedges or wax molds with predefined incremental values to determine the level of mouth opening [8,36]. Terebesi et al. used a hydrostatic device to fix the level of opening [5]. A similar system could reduce the error level observed in the present study, although it would be necessary to determine whether the presence of such a device modifies the behavior of the elevator muscles. Additionally, the method used should allow variable mouth opening increments to enable normalization, rather than fixed increments that are the same for all subjects.

The effect of normalization by comparing absolute values with normalized values is shown in Figure 2a–h. In the case of the right temporal muscle, subject 04F19 showed higher activity when the data were analyzed in absolute terms (Figure 2a); however, after normalization, it became evident that the right temporal muscle of subject 04F19 had muscle activity that fell within the range of the other subjects (Figure 2e). Regarding the left temporal muscle, subject 03M19 presented the highest absolute activity for MIC (Figure 2b), but once normalized, subject 02M22 showed the highest level of relative muscle activity at MIC (Figure 2f). For the masseter muscles, the effect of the normalization procedure was evident in subject 01F20. When considering absolute activity values (Figure 2c–d), this subject exhibited higher muscle activity compared to the other participants. However, after normalization, the activity level of subject 01F20 fell below that of subject 02M22 for the right masseter muscle (Figure 2g) and became comparable to the rest of the subjects for the left masseter muscle (Figure 2h).

With respect to the effect of normalizing mouth opening, a difference of 18 mm was observed between the lowest and the highest MMO (Table 1). This discrepancy is overcome through normalization.

One limitation of the EMG signal normalization method is that it may be constrained in patients with temporomandibular disorders, as pain may prevent them from performing MVC. In studies involving such patients, alternative approaches such as dynamic normalization methods should be considered [25]. Zieliński et al. [38] found that studies related to the temporomandibular joint and masticatory muscles require a sample size of 130 participants to detect a medium effect size (g = 0.3) with 80% statistical power. Based on this, the sample of four participants in the present study represents a major limitation when drawing conclusions from the results. This should be taken into account in future research addressing this field. To reduce variability in the results, surface electromyography requires careful planning regarding both the type of electrode and its placement. Electrodes should be placed in the most prominent area of the muscle, and they must be of an appropriate size to avoid interference from adjacent muscles in the signal acquisition. The distance between electrodes is also a factor to consider and should be fixed before starting the study [1,39]. This may limit the use of surface electromyography for the study of masticatory muscles if appropriately sized electrodes are not available.

For the right and left anterior temporal muscles, descending curves from MIC to a minimum point followed by an increase in activity were observed, as reported in the literature (Figure 2e–f) [5,7,8,9,36,37,40]. The mechanism explaining this behavior involves a combination of mechanical factors, where the elastic and viscoelastic elements of the elevator muscles help maintain the postural position along with proprioceptive control. Initially, active contraction maintains tooth contact, which explains the elevated initial activity. Immediately after mandibular separation, viscoelastic elements come into play, and the activation required to maintain the mandibular position tends to decrease until a minimum is reached. Meanwhile, the active muscle regulation mechanism activates the depressor muscles while inhibiting the elevator muscles [29]. Beyond a certain level of opening, due to muscle spindle stretching, the tonic stretch reflex is triggered, causing a slight contraction to counteract overstretching, peaking at MMO [6].

When analyzing the trend in muscle activity, the masseter muscles show an irregular pattern, with an initial increase followed by a decrease to a minimum and then an increase again at MMO (Figure 2g–h). Suvinen et al. reported similar behavior for masseter muscle activity in both healthy subjects (*n* = 15) and those with TMD (*n* = 18) [8]. Michelotti et al. reported similar behavior in a single subject, although when averaging the full sample (*n* = 40), the decreasing and then increasing pattern described in the rest of the literature was observed [7]. This may suggest a subject-specific behavior that becomes more prominent with smaller sample sizes. A feature not previously reported in the literature is the abrupt drop observed in the right temporal muscle of subject 04F19 (Figure 2f) and in the left masseter muscle of subject 01F20 upon reaching MMO (Figure 2h).

## 5. Conclusions

A protocol for the normalization of mouth opening and muscular activity using electromagnetic articulography and surface electromyography was successfully developed and applied. The results are consistent with the literature, showing elevated muscle activity at MIC, which tends to decrease up to a certain point and then increases again until MMO is reached. A limitation of this study is the small sample size, which prevented comparisons across different degrees of mouth opening to determine whether a point of minimal muscle activity exists. The muscle activity normalization method may not be appropriate for subjects with TMD.

## Figures and Tables

**Table 1:** The values used for the normalization of muscle activity.

Subject	V_MVC_—RT [µV]	V_MVC_—LT [µV]	V_MVC_—RM [µV]	V_MVC_—LM [µV]	MMO [mm]	∆5% [mm]
01F20	48.0	62.0	62.7	83.0	28.1	1.4
02M22	233.4	233.7	101.8	168.0	41.3	2.1
03M19	82.5	82.3	229.8	317.6	32.9	1.6
04F19	128.8	136.5	162.9	240.6	46.3	2.3

Note: RT, LT, RM, and LM correspond to right temporal, left temporal, right masseter, and left masseter muscles, respectively. VMVC: maximum voluntary clenching activity. MMO: maximum mouth opening.

## Data Availability

The data presented in this study are available on request from the corresponding author. The data are not publicly available due to no public database being available.

## Abbreviations

The following abbreviations are used in this manuscript:

EMA	Electromagnetic Articulography
sEMG	Surface Electromyography
MMO	Maximum Mouth Opening

## References

[1] Zieliński G., Gawda P. (2024). Surface Electromyography in Dentistry—Past, Present and Future. JCM.

[2] Suvinen T.I., Kemppainen P. (2007). Review of Clinical EMG Studies Related to Muscle and Occlusal Factors in Healthy and TMD Subjects. J. Oral Rehabil..

[3] Garnick J., Ramfjord S.P. (1962). Rest Position. J. Prosthet. Dent..

[4] Gross M.D., Ormianer Z., Moshe K., Gazit E. (1999). Integrated Electromyography of the Masseter on Incremental Opening and Closing with Audio Biofeedback: A Study on Mandibular Posture. Int. J. Prosthodont..

[5] Terebesi S., Giannakopoulos N.N., Brüstle F., Hellmann D., Türp J.C., Schindler H.J. (2016). Small Vertical Changes in Jaw Relation Affect Motor Unit Recruitment in the Masseter. J. Oral Rehabil..

[6] Okeson J.P. (2003). Management of Temporomandibular Disorders and Occlusion.

[7] Michelotti A., Farella M., Vollaro S., Martina R. (1997). Mandibular Rest Position and Electrical Activity of the Masticatory Muscles. J. Prosthet. Dent..

[8] Suvinen T.I., Reade P.C., Könönen M., Kemppainen P. (2003). Vertical Jaw Separation and Masseter Muscle Electromyographic Activity: A Comparative Study between Asymptomatic Controls & Patients with Temporomandibular Pain & Dysfunction. J. Oral Rehabil..

[9] Majewski R.F., Gale E.N. (1984). Electromyographic Activity of Anterior Temporal Area Pain Patients and Non-Pain Subjects. J. Dent. Res..

[10] Manns A., Miralles R., Santander H., Valdivia J. (1983). Influence of the Vertical Dimension in the Treatment of Myofascial Pain-Dysfunction Syndrome. J. Prosthet. Dent..

[11] Akbulut N., Altan A., Akbulut S., Atakan C. (2018). Evaluation of the 3 Mm Thickness Splint Therapy on Temporomandibular Joint Disorders (TMDs). Pain Res. Manag..

[12] Singh B.P., Singh N., Jayaraman S., Kirubakaran R., Joseph S., Muthu M.S., Jivnani H., Hua F. (2024). Occlusal Interventions for Managing Temporomandibular Disorders. Cochrane Database Syst. Rev..

[13] Nota A., Caruso S., Ehsani S., Ferrazzano G.F., Gatto R., Tecco S. (2021). Short-Term Effect of Orthodontic Treatment with Clear Aligners on Pain and sEMG Activity of Masticatory Muscles. Medicina.

[14] Woźniak K., Szyszka-Sommerfeld L., Lichota D. (2015). The Electrical Activity of the Temporal and Masseter Muscles in Patients with TMD and Unilateral Posterior Crossbite. BioMed. Res. Int..

[15] Ribeiro A.B., Pita M.S., Ribeiro A.B., Garcia A.R., Junqueira Zuim P.R. (2022). Effect of Short-Term Increase in Occlusal Vertical Dimension on Masticatory Muscle Electrical Activities and Pressure-to-Pain Threshold: A Crossover Clinical Study. J. Prosthet. Dent..

[16] Madhavan S., Dhanraj M., Jain A.R. (2018). Methods of Recording Mandibular Movements—A Review. Drug Invent. Today.

[17] Manns A., Miralles R., Cumsille F. (1985). Influence of Vertical Dimension on Masseter Muscle Electromyographic Activity in Patients with Mandibular Dysfunction. J. Prosthet. Dent..

[18] Shigemitsu R., Ogawa T., Sato E., Oliveira A.S., Rasmussen J. (2025). Kinematic Classification of Mandibular Movements in Patients with Temporomandibular Disorders Based on PCA. Comput. Biol. Med..

[19] Tian S., Dai N., Li L., Li W., Sun Y., Cheng X. (2020). Three-Dimensional Mandibular Motion Trajectory-Tracking System Based on BP Neural Network. Math. Biosci. Eng..

[20] Shiga H., Itoh Y., Yokoyama M., Komino M., Nakajima K., Kobayashi Y. (2022). Effect of Occlusal Interference on the Masticatory Movement Path. J. Jpn. Acad. Occulusion Health.

[21] D’Attilio M., Di Carlo B., Caroccia F., Moscagiuri F., d’Angelo D.M., Chiarelli F., Festa F., Breda L. (2021). Clinical and Instrumental TMJ Evaluation in Children and Adolescents with Juvenile Idiopathic Arthritis: A Case—Control Study. Appl. Sci..

[22] Zawawi K.H., Al-Badawi E.A., Lobo S.L., Melis M., Mehta N.R. (2003). An Index for the Measurement of Normal Maximum Mouth Opening. J. Can. Dent. Assoc..

[23] Hawwa M.M. (2022). Mouth Opening Range for Jordanian Population and Its Relation to Gender, Age, Height, and Weight. Saint’s Int. Dent. J..

[24] Diong J., Kishimoto K.C., Butler J.E., Héroux M.E. (2022). Muscle Electromyographic Activity Normalized to Maximal Muscle Activity, Not to Mmax, Better Represents Voluntary Activation. PLoS ONE.

[25] Sinclair J., Taylor P.J., Hebron J., Brooks D., Hurst H.T., Atkins S. (2015). The Reliability of Electromyographic Normalization Methods for Cycling Analyses. J. Hum. Kinet..

[26] Kulchutisin P., Sowithayasakul T., Pumklin J., Piyapattamin T. (2023). Electromyographic Evaluations of Masticatory Muscle Activity between Patients with Skeletal Class I and III Relationships. Eur. J. Dent..

[27] Ferrario V.F., Sforza C., Miani A., D’Addona A., Barbini E. (1993). Electromyographic Activity of Human Masticatory Muscles in Normal Young People. Statistical Evaluation of Reference Values for Clinical Applications. J. Oral Rehabil..

[28] Matsui M.Y., Giannasi L.C., Batista S.R.F., Amorim J.B.O., Oliveira C.S., Oliveira L.V.F., Gomes M.F. (2017). Differences between the Activity of the Masticatory Muscles of Adults with Cerebral Palsy and Healthy Individuals While at Rest and in Function. Arch. Oral Biol..

[29] Manns A., Miralles R., Guerrero F. (1981). The Changes in Electrical Activity of the Postural Muscles of the Mandible upon Varying the Vertical Dimension. J. Prosthet. Dent..

[30] Montero J., Castillo-Oyagüe R., Lynch C.D., Albaladejo A., Castaño A. (2013). Self-Perceived Changes in Oral Health-Related Quality of Life after Receiving Different Types of Conventional Prosthetic Treatments: A Cohort Follow-up Study. J. Dent..

[31] Schiffman E., Ohrbach R., Truelove E., Look J., Anderson G., Goulet J.-P., List T., Svensson P., Gonzalez Y., Lobbezoo F. (2014). Diagnostic Criteria for Temporomandibular Disorders (DC/TMD) for Clinical and Research Applications: Recommendations of the International RDC/TMD Consortium Network* and Orofacial Pain Special Interest Group. J. Oral Facial Pain Headache.

[32] Farfán N.C., Lezcano M.F., Navarro-Cáceres P.E., Sandoval-Vidal H.P., Martinez-Gomis J., Muñoz L., Marinelli F., Fuentes R. (2023). Characterization of Mandibular Border Movements and Mastication in Each Skeletal Class Using 3D Electromagnetic Articulography: A Preliminary Study. Diagnostics.

[33] Lezcano M.F., Dias F., Arias A., Fuentes R. (2020). Accuracy and Reliability of AG501 Articulograph for Mandibular Movement Analysis: A Quantitative Descriptive Study. Sensors.

[34] Szyszka-Sommerfeld L., Machoy M., Lipski M., Woźniak K. (2020). Electromyography as a Means of Assessing Masticatory Muscle Activity in Patients with Pain-Related Temporomandibular Disorders. Pain Res. Manag..

[35] Lindauer S.J., Gay T., Rendell J. (1993). Effect of Jaw Opening on Masticatory Muscle EMG-Force Characteristics. J. Dent. Res..

[36] Rugh J.D., Drago C.J. (1981). Vertical Dimension: A Study of Clinical Rest Position and Jaw Muscle Activity. J. Prosthet. Dent..

[37] Plesh O., McCall W.D., Gross A. (1988). The Effect of Prior Jaw Motion on the Plot of Electromyographic Amplitude versus Jaw Position. J. Prosthet. Dent..

[38] Zieliński G., Gawda P. (2025). Defining Effect Size Standards in Temporomandibular Joint and Masticatory Muscle Research. Med. Sci. Monit..

[39] Mehr K. (2013). Surface Electromyography in Orthodontics—A Literature Review. Med. Sci. Monit..

[40] Woda A., Pionchon P., Palla S. (2001). Regulation of Mandibular Postures: Mechanisms and Clinical Implications. Crit. Rev. Oral Biol. Med..

